# American Association of Obstetricians and Gynecologists

**Published:** 1894-10

**Authors:** 


BUFFALO MEDICAL AND SURGICAL JOURNAL.
A MONTHLY REVIEW OF MEDICINE AND SURGERY.

*EDITORS:*

THOMAS LOTHROP, M. D. - - WM. WARREN POTTER, M. D.

All communications, whether of a literary or business nature, should be addressed to the managing editor: 284 FRANKLIN STREET, BUFFALO, N. Y.

VOL. XXXIV. OCTOBER, 1894. No. 3.

### AMERICAN ASSOCIATION OF OBSTETRICIANS AND GYNECOLOGISTS.

THIS sturdy and progressive association held its seventh annual meeting in Toronto, Ont., September 19, 20, 21, 1894.

We speak quite within the bounds of truth when we assert that this meeting was a most satisfactory one to the association and its many friends, from any and every view-point taken. To begin with, the attendance of the Fellows was the largest in the history of the association. Then the audience itself, composed of guests and visitors, at every session was large, lending zest and spirit to the occasion. The invigorating air of Toronto, too, played an active part in stimulating the debaters to do good work. For example, after one day's hard work, an adjournment was had at 5 o'clock, P. M., to permit the association and its guests to enjoy a sail on the steam yacht Cleopatra, tendered by her owners, Messrs. A. E. Gooderham and T. G. Blackstock, after which refreshments were served at the Royal Yacht Club rooms, provided by one of the Fellows of the association, Dr. James F. W. Ross. The invigorating effect of this yachting cruise was exhibited in the animated debate on appendicitis, which opened the same evening at 8 o'clock, and lasted until midnight. Whoever witnessed this splendid exhibition of mental gymnastics could not fail to be impressed with the scene, as well as instructed by the eloquence and earnestness of the debaters. If general practitioners had been in doubt as to the time for operation in cases of appendicitis, such uncertainty must have been dispelled by the forceful manner in which Dr. J. B. Murphy, of Chicago, in one breath put and answered the question, "When shall we operate?" "Now," said Dr. Murphy, "not tomorrow, today, now" ! ! !

The second day, Thursday, the work began at 9 o'clock, and continued, with one hour's intermission, until 5.30 P. M. At 6.30 P. M. the Fellows assembled at the Toronto Athletic Club, and after the business meeting was concluded, the annual dinner was served. Covers were laid for fifty, and all the chairs were occupied. His Excellency, Mr. Fitzpatrick, lieutenant-governor of the province of Ontario, sat at the president's right, and Professor Goldwin Smith occupied the chair at his left.

Both of these gentlemen made clever speeches, the one in response to the Dominion of Canada, and the other to Sister Professions. Several bright and witty speeches were made, but the one in response to the Medical Profession of Canada, by Dr. Thomas J. Harrison, set the banqueters all aglow with mirth and good feeling. The speeches were frequently punctuated with music by the orchestra and songs by excellent male voices.

The third day opened with the usual spirit, and the work was carried on, with the exception of a short recess, until the final adjournment at 5 o'clock, P. M.

The profession of Canada turned out in full force to attend the several sessions, and there was no time when the chairs were not occupied to nearly the extent of their capacity. The impression left behind by the association was that it is a working body, having little or no time for frolic during the days of its official meeting.

Its annual dinner, on the evening of the second day, is the only recreation that it usually affords itself. On this occasion each Fellow pays for the service of his own plate, and is privileged to invite guests. This fashion was adopted so that the association might feel at liberty to appoint its annual meeting in any city it chose without being a tax on the local profession where it meets for entertainment, as it accepts no hospitalities that either interfere with its work or makes it onerous to the physicians of the city of its meeting. In other words, it desires to feel free to meet whereever it prefers, pay its own way and go about its work in a professional fashion.

The resident Fellows in Toronto, who constituted the local committee of arrangements, are entitled to great praise for the efficient manner in which they conducted the affairs of the meeting. The Council Chamber of the College of Physicians and Surgeons of Ontario, where the association met, is an ideal place in which to hold meetings of this kind. Its acoustic properties are perfect and no street noise interferes with the debaters. At the conclusion of the meeting a vote of thanks was tendered to Dr. James F. W. Ross, chairman; Dr. A. H. Wright, Dr. I. H. Cameron, treasurer; Dr. R. B. Nevitt and Dr. H. S. Machell for the efficient manner in which they had performed the duties as committee of arrangements.

The officers elected for the ensuing year are: President, Dr. J. Henry Carstens, Detroit; Vice-Presidents, Drs. W. E. B. Davis, Birmingham, Ala., and Henry Howitt, Guelph, Ont.; Secretary, Dr. William Warren Potter, Buffalo; Treasurer, Dr. X. O. Werder, Pittsburg; Executive Council: Dr. Charles A. L. Reed, Cincinnati; Dr. James F. W. Ross, Toronto; Dr. Joseph Price, Philadelphia; Dr. A. Vander Veer, Albany; and Dr. L. S. McMurtry, Louisville.

From Buffalo two new Fellows were elected—namely, Drs. Herman E. Hayd and Stephen Y. Howell. The next place of meeting is Louisville, Ky., and the time chosen is during the third week in September, 1895.

